# Predicting schoolchildren’s willingness to eat insect-based snacks: effects of information provision psychological and cognitive traits

**DOI:** 10.1038/s41538-026-00826-3

**Published:** 2026-04-08

**Authors:** Jatziri Mota-Gutierrez, Lotte Pater, Sabien Journée, Manouk Grevelman, Karthika Srikanthithasan, Claudio Forte, Paola Toschi, Maryia Mishyna

**Affiliations:** 1 Department of Veterinary Sciences, University of Turin, Largo Paolo Braccini 2, Turin, 10095 Turin Italy; 2 Food Quality and Design Group, Wageningen University and Research, P.O. Box 17, Wageningen, 6700 The Netherlands

**Keywords:** Health care, Psychology, Psychology

## Abstract

Disgust and neophobia limit insect-food acceptance, while education has variable effects on entomophagy attitudes. This study examined how food neophobia, disgust, educational information, entomophagy awareness, and eating behaviour profiles influence Italian children’s (8–10 years old) acceptance of insect-based foods and how these traits moderate educational effects. Children participated in two in-person lectures and completed questionnaires before and after, featuring images of buffalo worms, crickets, and protein bars and chocolates containing these insects. Results showed that educational information increased willingness to eat insect-based foods. Children low in food neophobia and disgust were more likely to accept these products, and educational information reduced the negative influence of neophobia, particularly for cricket protein bars. These findings indicate that targeted education can partially offset reluctance. It offers practical guidance for snack producers, healthcare professionals, and policymakers to promote sustainable, nutritious eating in children by addressing psychological barriers and fostering openness to novel foods.

## Introduction

By 2050, the world’s population is expected to reach 9.7 billion people^1^. This fast-growing world population increases food demand and causes growing pressure on the world’s resources and environment. To meet future needs, a transition towards a sustainable food system is highly needed, with alternative protein sources replacing the conventional livestock proteins^2^. An example of such promising alternative proteins is edible insects^3^. Edible insects have high levels of protein and essential amino acids, and are a good source of fibre, mono- and polyunsaturated fats, minerals, and vitamins^4^. In addition to these health benefits, insects have high feed conversion efficiency and have significantly less environmental impact^5^. A study by Olivadese and Dindo highlighted that while entomophagy, the consumption of insects, was once practiced in Western societies, it gradually declined due to cultural biases and the development of alternative food sources^6^. Over time, the consumption of insects has become increasingly uncommon in Western society and is now generally regarded as unconventional. Insects often provoke negative reactions in adults, who perceive them as dirty, disgusting, or dangerous^7,8^.

Research on the acceptance of edible insects primarily focuses on adults, leaving entomophagy acceptance among children under researched^1,2,9^. This is striking, as children represent a distinct group whose entomophagy acceptance cannot be assumed to mirror that of adults. This is in line with the review by Kröger et al., who note that existing research on how age influences the acceptance of edible insects is fragmented^10^. They emphasize the need for more studies to determine whether younger people are indeed more open to insect-based foods, as this would offer valuable insights for developing age-specific outreach and marketing strategies.

It can be assumed that children’s acceptance differs from that of adults for three reasons. First, children have higher levels of food neophobia and respond stronger to unfamiliar foods than adults^11^, which may influence their willingness to consider insects as edible. Second, childhood represents a formative period during which attitudes begin to develop. Children’s food preferences are still forming, making them more sensitive to early experiences with novel foods. The socialisation of what food is “good” and “bad” food starts during this childhood^12^. According to Maya et al., entomophagy in children shows varying results across different ages (4-to-19-year-old children) and Western European cultures (Sweden, Denmark, France, Germany, and the Netherlands)^2^. Cultural food diets evolve over generations through economic and sociocultural changes, allowing young consumers to adopt new food habits that may be unfamiliar to previous generations. Third, children’s eating behaviours are influenced by parents, peers, and institutional settings such as schools. These social environments can influence openness to novel foods, such as insects. Adults, on the other hand, typically make independent food choices. The mechanisms driving acceptance are therefore not comparable to adults^13^.

Limited research has been done on children’s acceptance of edible insects, which suggests that a variety of factors shape their willingness to try insect-based foods. Psychological factors, such as food neophobia and food disgust, play an important role. Food neophobia is considered a psychological trait that often leads individuals to eat a limited range of foods and to be less willing to try new or sustainable options, such as insect-based foods. Unlike food neophobia, which is rooted in unfamiliarity, food disgust is predominantly driven by disgust reactions toward sensory characteristics such as texture, odour, and appearance^14^. Studies indicate that food neophobia lowers the hedonic rating, willingness to try, or acceptance of insects or insect-based foods^1,2,15,16^. Children perceive grasshoppers, mealworms, and crickets as scary and something that one is not allowed to eat^12^. Moreover, insects are associated by children with dirt and poverty^17^. Despite these findings on feelings of disgust, Danish children showed that disgust is not correlated with the willingness to try insects^1^. Besides food neophobia and food disgust, high awareness of insects as food in general increases the willingness to consume them^16^. Given that these studies are based on a mix of quantitative and qualitative data, with most of these studies based on quantitative data and only two based on qualitative data, their findings may not be readily generalizable to larger populations. Consequently, integrating the key factors influencing the acceptance of edible insects offers valuable insight into their interrelationships.

Building on these psychological influences, children’s reactions also vary depending on the specific insect species and how visibly the insects are incorporated into the food. Regarding the type of insect, Erhard et al. found that the hedonic ratings of the cricket were rated lower by children than the buffalo worm, potentially due to the higher perceived animalness of the cricket^1^. A deeper understanding of how eating behaviours shape children’s acceptance and future consumption of edible insect-based foods can help smooth the transition towards the consumption of insects as an alternative protein.

The limited focus on children in research is striking, as children can play a pivotal role in the acceptance of edible insects. Children shape family food choices and influence their peers’ preferences, as a result, potentially increasing entomophagy acceptance in their social environment. Children familiar with eating edible insects are more likely to include these products in their diets as adults^15,17^. However, understanding how to engage children is crucial for the long-term adoption of edible insects^17^. Previous research with adults suggests that information provision about health, taste, and sustainability could reduce the fear of trying edible insects, although results are mixed^10^. Information on the benefits of eating insect-based food, such as healthiness and sustainability benefits, could influence consumers’ perception^18^. For children, early education can enhance the acceptance of edible insects later in life, as childhood eating habits often persist into adulthood. The acceptance of edible insects among children can be increased by providing information about the possibilities of eating edible insects and ways of preparing them^12^. Although tactic interaction with insects is argued as a method to increase acceptance of edible insects amongst 8- to 13-year-old French children^17^, Chow et al. showed that cooking with insects does not increase the liking of insects amongst 11- to 13-year-old Danish children, potentially due to levels of children’s disgust^15^. Alternatively, Nyberg et al. argue that providing information about the edibility of insects as well as talking about insects in a more positive way can increase their acceptance amongst 4- to 5-year-old Swedish children^12^.

Yet the effectiveness of such positive framing is influenced by the fact that children typically have little knowledge of the nutritional or ecological benefits of insects^17^. Therefore, basic information about environmental and health benefits should be provided to promote consumption^16^. By providing this education in a school context, the acceptance of edible insects can be stimulated even further^12^. Being in a social group can help overcome children’s barriers as the group context helps to transform the novel food experience into a fun challenge^17^. The effects of educating children about the health and environmental benefits of insects in school settings remain largely unexplored. Gaining insight into how health and environmental information about insects influences children in school settings is essential for developing effective educational strategies that promote sustainable food choices from an early age. Moreover, recent studies have shown that age, cultures, food neophobia, food disgust, and awareness of edible insects are all determinants of children’s attitudes or perceptions towards edible insects^2,12,15–17^.

By integrating both psychological and cognitive trait predictors (food neophobia, food disgust, eating behaviours, and entomophagy awareness) and educational interventions, this study aims to identify the direct influence of these traits on edible insect-based foods’ acceptance and the moderating role of these traits on educational interventions. This integrated approach is important as it allows to identify which subgroups of children are most responsive to education, providing practical implications for designing interventions to increase acceptance of insect-based foods. To achieve this, an interactive learning activity, two lectures, and a survey were developed and carried out in primary schools in Turin, Italy. The food neophobia, food disgust, entomophagy awareness, willingness to eat, and acceptance of buffalo worm and cricket protein bars and chocolates were measured using images. The selected types of insects are available in the Italian market, while the selected types of snacks are the most consumed food products in European children’s diet^19^. Italy presents an interesting case for studying children’s acceptance of insect-based foods due to its strong culinary traditions. While Italian cuisine is traditionally resistant to novel food sources, some products already incorporate insects^20^, such as Sardinia’s Casu Marzu cheese, which contains live insect larvae and holds cultural significance^21^. This suggests that while there is resistance, there is also potential for insect-based foods to be embraced in Italy. Special attention was given to identify eating behaviour profiles among Italian children. These clusters were characterized according to: (a) their food neophobia, (b) their level of awareness towards entomophagy, and (c) food disgust. Further, these clusters were used to predict acceptance towards insect-based snacks.

Based on the literature and the aims of this study, we formulated the following hypotheses:

H1. Educational information increases children’s willingness to eat and acceptance of insect-based foods, and its effectiveness may vary depending on children’s individual traits.

H2. Children with higher levels of food neophobia or food disgust decrease their willingness to eat and acceptance of insect-based foods.

H3. Children’s eating-behaviour profiles influence acceptance of insect-based foods.

The findings of the present study offer valuable insights for the growing insect-based foods industry and help shed light on the key factors that influence food acceptance or rejection among schoolchildren.

## Results

A total of 53% of the participating children were females, and 48% were 9-year-old children, 91% lived with both parents, and in 85% of cases, both parents were employed (Table 1). More than half of the participants reported awareness of the concept of insects as food. Only 20 participants (9.01%) reported having tasted insects before. Based on the total score of the Food Neophobia Test Tool (FNTT) and Food Disgust Scale (FDS), more than half of the children showed a moderate food neophobia and high food disgust.

**Table 1:** Socio-demographic and personal characteristics of the sample (*n* = 222)

	Counts (*N*)	Percentage (%)
Gender		
Females	117	52.70
Males	105	47.30
Age		
8	69	31.08
9	95	42.79
10	58	26.13
Living situation		
Both parents	202	91.89
Father	3	1.35
Mother	14	0.45
Tutor	2	0.90
Parents occupation		
Both	189	85.14
Father	24	10.81
Mother	3	1.35
Not to say	6	2.70
Entomophagy awareness		
No awareness	48	20.72
Aware but not eating inexperience	155	69.82
Awareness and eating experience	20	9.01
Food neophobia		
Neophobic/High	25	11.26
Moderate	125	56.31
Neophilic/Low	72	32.43
Food disgust		
Low	4	5.41
Moderate	64	27.93
High	146	65.77

Respondents were assigned to the level of neophobia groups based on the sum of the scores for the items in the food neophobia test tool: “neophilic” (subjects in the lowest quartile, FNTT ≤ 25), “moderate” (subjects in the second and third quartile, FNTT ≥ 26 and ≤ 35), and “neophobic” (subjects in the highest quartile, FNTT ≥ 36). Respondents were assigned to the level of food disgust based on the sum of the scores for the items in the food disgust scale: “low” (subjects in the lowest quartile, FDS ≤ 18), “moderate” (subjects in the second and third quartile, FDS ≥ 19 and ≤ 28), and “high” (subjects in the highest quartile, FDS ≥ 29).

FNTT scores ranged from 16 (low scores—neophilic) to 50 (high scores—neophobic). The total sample (*n* = 222) had a mean of 32.28 ± 5.16. There were no significant gender, age, or intervention differences with respect to FNTT (*P* = 0.47, *P* = 0.65 and *P* = 0.66, respectively). FDS scores ranged from 16 (low disgust sensitivity) to 40 (high disgust). The total sample had a mean of 30.60 ± 4.64. Females scored higher (*P* = 0.002) on the FDS (31.02 ± 5.23) than males (29.51 ± 5.91). There were no significant age or intervention differences with respect to FDS (*P* = 0.32 and *P* = 0.67, respectively). The total sample score of awareness towards edible insect products had a mean of 1.91 ± 0.56. There were no gender or intervention differences with respect to entomophagy awareness (*P* = 0.28 and *P* = 0.47, respectively), regardless of their age.

### Predictors and moderation of children’s willingness to eat a snack made from insects

There was no difference between females and males in their willingness to eat (WTE) a snack made from insects, either before (*P* = 0.31) or after (*P* = 0.42) the intervention, regardless of age (9-year-old *P* = 0.31, and 10-year-old *P* = 0.10, respectively). The Cumulative Link Mixed Model (CLMM) revealed that educational information, neophobia and food disgust are significant predictors for WTE of insect-based snacks (Table 2). Further, moderation analysis indicated that the effect of educational interventions on children’s WTE insect-based snacks was significant only for those with neophilic (low FNTT scores) and moderate food neophobia. In contrast, no interaction effects emerged for food disgust or prior entomophagy awareness (Table 2).

**Table 2:** Parameters estimates from the cumulative link mixed model, including main effects and interaction terms, predicting children’s willingness to eat an insect-based snack (*n* = 222)

Predictors	Estimate	SE	*Z*-value	*P*-value
Educational information	1.6856	0.2100	7.948	<0.001
Educational information moderation				
E_Information:FDS_Low	−0.6898	11.4930	−0.600	0.5484
E_Information:FDS_Moderate	0.0676	0.4226	0.160	0.8729
E_Information:FNTT_Neophobic	−0.1970	0.4324	−0.456	0.6486
E_Information:FNTT_Neophilic	−16.324	0.7596	−2.149	0.0316
Food Neophobia Test Tool				
FNTT_Neophobic	1.2185	0.2391	5.097	<0.001
FNTT_Neophilic	−1.9742	0.4248	−4.647	<0.001
Food Disgust Scale				
FDS_Low	0.9430	0.5567	1.694	0.0903
FDS_Moderate	0.5645	0.2342	2.41	0.0159

*FNTT* Food neophobia test tool, *FDS* Food disgust scale, *SE* Standard error.

**Fig. 1:**
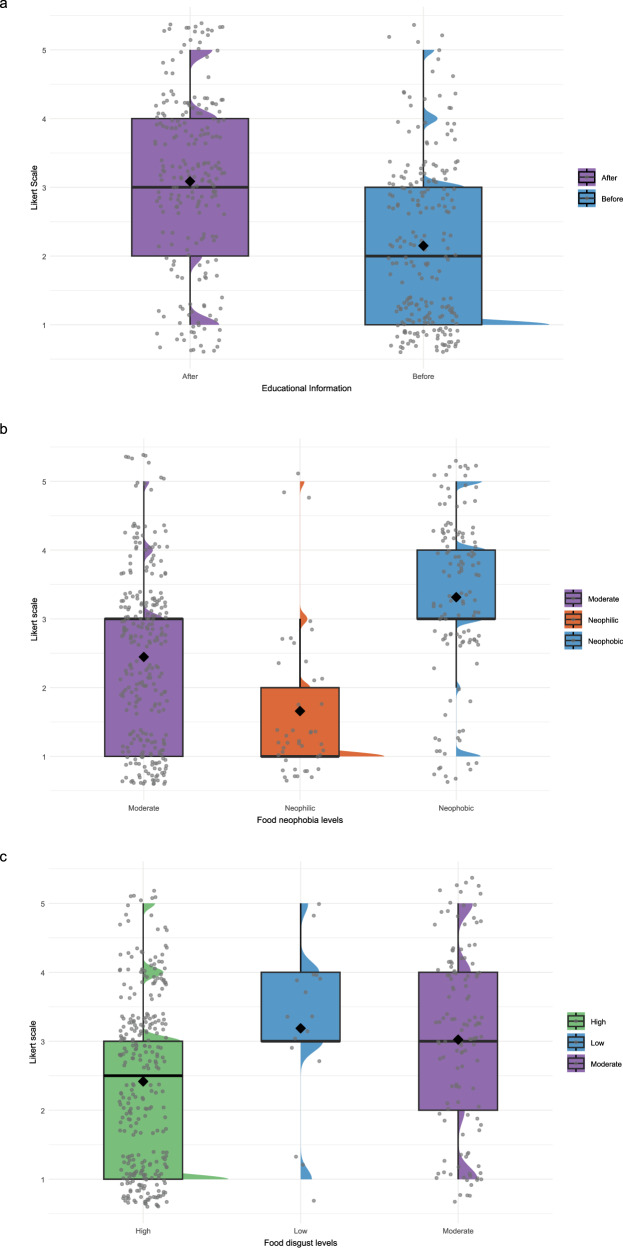
Raincloud plot showing individual responses and mean willingness to eat a snack made from insects by Italian children (children = 222; survey = 444). Results derived from a cumulative link model controlling for food neophobia levels (**b**) food disgust levels (**c**) and educational information (**a**) towards willingness to eat a snack made from insects. Willingness to try scale: 1 = absolutely no to 5 = absolutely yes.

For food neophobia, neophilic children (low FNTT scores) had significantly higher WTE insect-based snacks, while highly neophobic children (High FNTT scores) had significantly lower WTE compared to the moderate reference group. Regarding food disgust, children with moderate disgust scores showed a significant positive association with WTE, whereas the effect for low disgust scores was not statistically significant compared with high disgust scores (Table 2). Overall, educating children about the health and environmental benefits of entomophagy (after educational information), as well as low levels of food disgust, and high levels of food neophobia (neophobic), were associated with significantly increased WTE insect-based snacks (Fig. 1).

### Predictors and moderation of children’s acceptance towards whole insects and insect-based snacks

The mean appropriateness of using buffalo worm or cricket as a food ingredient was 3.08 ± 1.41 and 3.17 ± 1.45, respectively. There were no significant gender or age differences with respect to the appropriateness of using both insects as food ingredients (*P* = 0.60 and *P* = 0.96 for buffalo worm and *P* = 0.64 and *P* = 0.50 for cricket). Mean WTE scores for whole buffalo worm and cricket were 2.62 ± 1.26 and 1.31 ± 2.39, respectively. Males scored significantly higher (*P* = 0.001 and *P* = 0.000, respectively) on the WTE whole buffalo worm and cricket (2.90 ± 1.31, and 2.72 ± 1.40, respectively) than females (2.37 ± 1.15, and 2.09 ± 1.15, respectively). Ten-year-old participants scored higher (*P* = 0.01 and *P* = 0.04, respectively) on the WTE whole buffalo worm and cricket (2.78 ± 1.22 and 2.50 ± 1.36, respectively) than eight-year-old (2.48 ± 1.30 and 2.26 ± 1.27, respectively).

Concerning the mean WTE buffalo worm protein bar was 2.39 ± 1.32, and the cricket protein bar was 2.28 ± 1.28. Ten-year-old participants scored higher (*P* = 0.04) on the WTE buffalo worm protein bars (31.02 ± 5.23) than eight-year-olds (29.51 ± 5.91). There were no significant gender or age differences with respect to WTE cricket protein bar (*P* = 0.06 and *P* = 0.38, respectively). In addition, the mean WTE buffalo worm and cricket chocolate was 2.66 ± 1.41 and 1.99 ± 1.19, respectively. Males scored significantly higher (*P* = 0.004) on the WTE cricket chocolate (2.21 ± 1.28) than females (1.78 ± 1.05). Ten-year-old participants scored higher (*P* = 0.04) on the WTE buffalo worm chocolate (2.91 ± 1.28) than eight-year-old (2.58 ± 1.45).

**Fig. 2:**
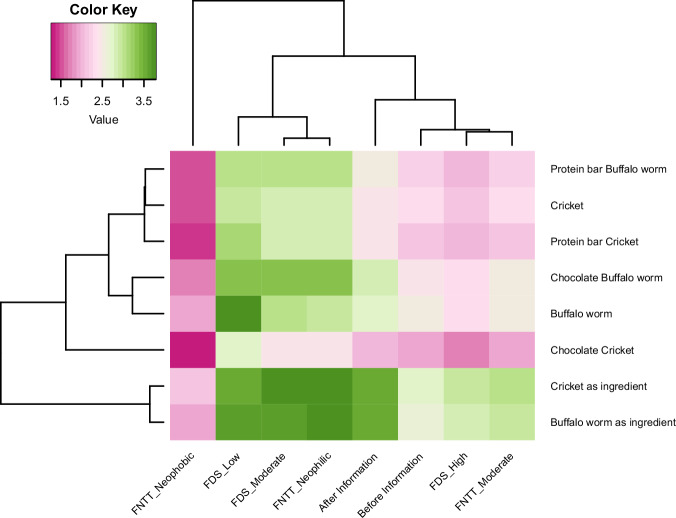
Acceptance of insects as food and ingredients among Italian children. Heatmaps of the levels of acceptance of insects as food (whole insects—buffalo worm and cricket, and food products—protein bars and chocolate) and appropriateness of using insects as food ingredients, and educational information, Food Neophobia Test Tool (FNTT), and Food Disgust (FDS) by Italian children (children = 222; surveys = 444). The intensity of the colours represents the mean values of the levels of acceptance of insects as food (whole insects—buffalo worm and cricket, and food products—protein bars and chocolate) and appropriateness of using insects as ingredients, and educational information, Food Neophobia Test Tool (FNTT) and Food Disgust (FDS). Acceptance scale of insects as food: 1 = very bad to 5 = very good. Acceptance scale of insects as ingredients: 1 = absolutely no to 5 = absolutely yes.

Figure 2 shows that acceptance of whole insects (buffalo worm and cricket), insect-based foods (protein bars and chocolate) and appropriateness of using insects as food ingredients varied across children, with clear differences between the relevant reference groups, such as those with moderate versus high levels of neophobia or disgust. Neophobic children had lower acceptance scores for all snacks. A strong disgust effect was also observed for all foods (except insects as ingredients). Children with low or moderate disgust sensitivity reported higher acceptance scores for all foods. Moreover, after educational provision also showed higher scores for insect-based snacks with respect to before the sessions. Overall, higher acceptance scores were also observed for the perceived appropriateness of buffalo worm and cricket as food ingredients (Fig. 2).

Tables 3 and 4 show educational information, food neophobia, and food disgust as significant predictors for acceptance of whole buffalo worm, buffalo worm protein bar, buffalo worm chocolate and the perceived appropriateness of buffalo worm and cricket as ingredients. Moderation analysis indicated that the effect of educational interventions on children’s acceptance of cricket protein bar was significant only for those with moderate and low food neophobia (neophilic), but the intervention effect was weaker for neophilic children relative to moderate (Table 3). No significant moderation was found for food disgust or food neophobia in the other foods (Table 3).

**Table 3:** Parameters estimates from the cumulative link mixed model, including main effects and interaction terms predicting children’s acceptance of buffalo worm or cricket snacks (children = 222; surveys = 444)

	Whole buffalo worm				Buffalo worm protein bar				Buffalo worm chocolate			
Predictor	Estimate	SE	*Z*-value	*P*-value	Estimate	SE	*Z*-value	*P*-value	Estimate	SE	*Z*-value	*P*-value
Educational information	0.4454	0.1885	2.362	0.0182	0.5300	0.1928	2.749	0.006	0.54023	0.19064	2.834	0.0046
Educational information moderation												
E_Information:FDS_Low	−0.3101	11.740	−0.264	0.7917	0.4342	11.7370	0.370	0.7114	−0.1366	12.513	−0.109	0.9131
E_Information:FDS_Moderate	0.2895	0.4311	0.672	0.5018	−0.4582	0.4326	−1.059	0.2896	−0.1500	0.4341	−0.345	0.7298
E_Information:FNTT_Neophobic	0.7146	0.4536	1.575	0.1150	0.6204	0.4630	1.340	0.1802	0.0736	0.5180	0.142	0.8870
E_Information:FNTT_Neophilic	−0.5312	0.7060	−0.752	0.4520	−0.9091	0.7815	−1.163	0.2447	−0.3668	0.6661	−0.551	0.5819
Food Neophobia Test Tool												
FNTT_Neophobic	0.3482	0.2601	1.338	0.1807	1.2723	0.26602	4.783	<0.001	1.1326	0.2629	4.309	<0.001
FNTT_Neophilic	−1.0833	0.4076	−2.658	0.0079	−1.0704	0.42297	−2.531	0.0114	−1.2797	0.4231	−3.025	0.0025
Food Disgust Scale												
FDS_Low	2.1658	0.6232	3.475	0.0005	1.3401	0.6304	2.126	0.0335	1.3844	0.6262	2.211	0.0270
FDS_Moderate	0.8830	0.2621	3.369	0.0008	1.0890	0.2567	4.242	<0.001	0.9380	0.2546	3.684	0.0002
	Whole cricket				Cricket protein bar				Cricket chocolate			
Educational information	0.1201	0.19034	0.631	0.5282	0.3361	0.1907	1.763	0.0779	−0.0475	0.1934	−0.245	0.8061
Educational information moderation												
E_Information:FDS_Low	0.8506	1.2477	0.682	0.4954	0.1632	11.398	0.143	0.8861	0.5903	113.5080	0.520	0.6031
E_Information:FDS_Moderate	0.0100	0.4401	0.023	0.9819	−0.3221	0.4343	−0.742	0.4582	−0.2692	0.4315	−0.624	0.5327
E_Information:FNTT_Neophobic	0.4201	0.4518	0.930	0.3524	0.7721	0.4575	1.688	0.0915	0.6183	0.4429	1.396	0.1627
E_Information:FNTT_Neophilic	−0.4375	0.7819	−0.560	0.5757	−26.314	0.9236	−2.849	0.0043	−0.8458	0.9057	−0.934	0.3504
Food Neophobia Test Tool												
FNTT_Neophobic	0.5746	0.2679	2.145	0.0319	1.0559	0.2562	4.122	<0.001	0.7648	0.2514	3.042	0.0024
FNTT_Neophilic	−1.4331	0.4395	−3.261	0.0011	−1.7674	0.4785	−3.693	0.0002	−1.8429	0.4993	−3.691	0.0002
Food Disgust Scale												
FDS_Low	0.8943	0.6422	1.393	0.1637	1.6417	0.5941	2.763	0.0057	1.4053	0.5910	2.378	0.0174
FDS_Moderate	0.6690	0.2604	2.569	0.0102	0.7992	0.2556	3.127	0.0018	0.7494	0.2445	3.065	0.0022

Results derived from a cumulative link model controlling for food neophobia, food disgust, and intervention towards willingness to eat a whole insects and insect-based products. Attitude scale “*How do you feel about the idea of looking or tasting the*…”: 1 = Very bad, 2 = Bad, 3 = Neutral, 4 = Good, 5 = Very good.

*FNTT* Food neophobia test tool, *FDS* Food disgust scale.

**Table 4:** Parameters estimates from the cumulative link mixed model, including main effects and interaction terms, predicting children’s appropriateness of using buffalo worm or cricket as food ingredients using a cumulative link mixed model (children = 222; surveys = 444)

	Buffalo worm as ingredient				Cricket as ingredient			
Predictor	Estimate	SE	*Z*-value	*P*-value	Estimate	SE	*Z*-value	*P*-value
Educational information	1.6239	0.2139	7.593	<0.001	1.3796	0.2045	6.747	<0.001
Educational information moderation								
E_Information:FDS_Low	0.1493	12.4560	0.120	0.9046	11.0370	11.931	0.925	0.3549
E_Information:FDS_Moderate	0.6513	0.4355	1.495	0.1348	0.6389	0.4442	1.438	0.1504
E_Information:FNTT_Neophobic	−0.3328	0.4400	−0.756	0.4493	−0.4483	0.4411	−1.016	0.3095
E_Information:FNTT_Neophilic	−11.504	0.7662	−1.502	0.1332	−0.1664	0.7424	−0.224	0.8226
Food Neophobia Test Tool								
FNTT_Neophobic	0.8487	0.2483	3.418	<0.001	0.6933	0.2465	2.813	<0.001
FNTT_Neophilic	−1.7069	0.4324	−3.947	<0.001	−1.4837	0.4121	−3.6	<0.001
Food Disgust Scale								
FDS_Low	1.4421	0.6292	2.292	<0.001	0.8928	0.578	1.545	0.1224
FDS_Moderate	0.8190	0.2493	3.285	<0.001	0.8226	0.2513	3.274	<0.001

Results derived from a cumulative link model controlling for food neophobia, food disgust, and intervention towards willingness to eat whole insects and insect-based products. Attitude scale *“Do you think that… can be used as ingredients”:* 1 = Absolutely no, 2 = No, 3 = Uncertain, 4 = Yes, 5 = Absolutely yes.

*FNTT* Food neophobia test tool, *FDS* Food disgust scale.

For food neophobia, neophilic children reported markedly higher acceptance of all insect-based snacks (except whole buffalo worm), whereas highly neophobic children showed significantly reduced acceptance relative to the moderate group. Children with lower and moderate disgust levels show much higher acceptance of all insect-based snacks (except whole crickets) compared to the high-disgust group (Table 3).

Educating children about the health and environmental benefits of entomophagy, as well as low levels of food disgust, was associated with significantly increased acceptance of insects as food and greater perceived appropriateness of their use as food ingredients (Table 4). In contrast, neophobic participants were significantly more likely to express rejection towards this type of food, as shown in Table 4.

### Eating behaviour profiles predict children’s acceptance of insect-based foods

Results from the latent class analysis (LCA) allowed to identify two classes of children based on their eating behaviour profiles with proportions for the latent classes of 50% (*n* = 223), 50% (*n* = 221), respectively, as shown in Table 5 based on the estimated model. The model showed classification of individuals with high certainty (low entropy = 0.1432) and about 79% of the maximum possible class separation achieved.

**Table 5:** Conditional item response probabilities of food neophobia, food disgust, and entomophagy awareness for two nutritional behaviour profiles (*n* = 444)

	Response	Cluster 1	SE	Cluster 2	SE
Cluster size		0.5023		0.4977	
Food neophobia					
I like to try foods I have never tasted before					
	Disagree	0.1602	0.0835	0.0002	0.0838
	Agree	0.3649	0.2741	0.9203	0.2734
	Uncertain	0.4749	0.1945	0.0795	0.1940
I like to experience new and different foods					
	Disagree	0.1783	0.0940	0.0000	0.0933
	Agree	0.3895	0.2762	0.9407	0.2750
	Uncertain	0.4322	0.1868	0.0593	0.1854
I think it is fun to try food items I don’t know					
	Disagree	0.3120	0.1603	0.0000	0.1592
	Agree	0.3616	0.2351	0.8325	0.2325
	Uncertain	0.3263	0.0858	0.1675	0.0830
I will try food even though I don’t know what it is					
	Disagree	0.6324	0.2289	0.1872	0.2273
	Agree	0.1035	0.1621	0.4178	0.1620
	Uncertain	0.2641	0.0778	0.3950	0.0746
I enjoy a wide variety of foods					
	Disagree	0.1977	0.1040	0.0121	0.0969
	Agree	0.5879	0.1666	0.9110	0.1610
	Uncertain	0.2144	0.0715	0.0769	0.0693
I am not afraid of eating things I have not tasted or experienced before					
	Disagree	0.4747	0.2321	0.0296	0.2284
	Agree	0.1753	0.3030	0.7861	0.3028
	Uncertain	0.3500	0.0865	0.1843	0.0871
I don’t mind eating foods I am not used to					
	Disagree	0.4004	0.1403	0.1328	0.1386
	Agree	0.3248	0.1517	0.6061	0.1471
	Uncertain	0.2748	0.0402	0.2611	0.0382
I think unfamiliar food looks unappetizing					
	Disagree	0.5574	0.0545	0.4778	0.0565
	Agree	0.2056	0.0440	0.2635	0.0447
	Uncertain	0.2370	0.0340	0.2587	0.0339
I am afraid of trying food I have not tasted before					
	Disagree	0.5516	0.0843	0.4018	0.0854
	Agree	0.2109	0.0746	0.3400	0.0762
	Uncertain	0.2375	0.0346	0.2582	0.0334
I am willing to taste foods made with insects					
	Disagree	0.8433	0.0520	0.7776	0.0472
	Agree	< 0.001	< 0.001	< 0.001	< 0.001
	Uncertain	0.1567	0.0520	0.2224	0.0472
Food disgust					
To eat with dirty silverware in a restaurant					
	Pleasant	0.0265	0.0352	0.0549	0.0249
	Disgusting	0.9488	0.0731	0.8429	0.0630
	Uncertain	0.0247	0.0446	0.1022	0.0433
Food donated from a neighbour whom I barely know					
	Pleasant	0.1515	0.0655	0.2550	0.0607
	Disgusting	0.4742	0.0746	0.3579	0.0730
	Uncertain	0.3742	0.0391	0.3872	0.0390
To eat hard cheese from which mold was cut off					
	Pleasant	0.1876	0.0603	0.2682	0.0518
	Disgusting	0.6980	0.0909	0.5618	0.0808
	Uncertain	0.1143	0.0412	0.1700	0.0395
To eat apple slices that turned brown when exposed to air					
	Pleasant	0.1409	0.0975	0.2977	0.0855
	Disgusting	0.6781	0.1225	0.4774	0.1112
	Uncertain	0.1810	0.0422	0.2249	0.0398
The texture of some kinds of fish in the mouth					
	Pleasant	0.2076	0.1160	0.4207	0.1107
	Disgusting	0.4806	0.1208	0.2649	0.1149
	Uncertain	0.3117	0.0371	0.3144	0.0349
To eat brown-coloured avocado pulp					
	Pleasant	0.0764	0.0792	0.1997	0.0712
	Disgusting	0.6927	0.0922	0.5444	0.0855
	Uncertain	0.2309	0.0370	0.2559	0.0343
There is a little snail in the salad that I wanted to eat					
	Pleasant	0.0543	0.0689	0.1721	0.0643
	Disgusting	0.8841	0.0926	0.7359	0.0847
	Uncertain	0.0615	0.0319	0.0919	0.0283
To put animal tendons into my mouth					
	Pleasant	0.1004	0.0484	0.1615	0.0428
	Disgusting	0.7857	0.0991	0.6406	0.0888
	Uncertain	0.1139	0.0592	0.1978	0.0550
Entomophagy awareness					
How much do you know about insects as food?					
	No awareness	0.1988	0.0310	0.2067	0.0282
	Awareness but not eating experience	0.6963	0.0349	0.6910	0.0324
	Awareness and eating experience	0.1049	0.0232	0.1023	0.0213

Akaike Information Criterion: 22850.94, Bayesian Information Criterion: 23526.75, Likelihood Ratio: 17107.85, Chi-square goodness of fit: 1.0405 × 10^15^, Entropy (average per subject): 0.1432, Relative entropy *R*^2^: 0.7933. *SE* Standard Error. Respondents were assigned to groups based on the scores of each item in the scales. Scores equal to or less than 2 were considered to indicate disagreement or pleasant; values greater than 3 were considered to indicate agreement or disgust, while 3 values were considered to indicate uncertainty.

Two distinct eating behaviour profiles were identified among children. The “*Cautious eaters”* (cluster 1) were characterized by lower openness to novel foods and were more likely to find food-related scenarios disgusting. In contrast, “*Adventures tasters*” (cluster 2) were characterized by a willingness to try unfamiliar foods, somewhat less disgusted by challenging food items and situations. In detail, *Cautious eaters* showed higher probabilities of disagreement or uncertain responses to positive statements and higher disgusting responses overall, especially for hygiene-based or visually unpleasant items (snails, tendons). While *Adventures tasters* showed a higher probability of agreement for all exploratory food items and were slightly more tolerant overall, but still often responded with disgust to some items (Table 5). Concerning entomophagy awareness, both clusters report similar levels of awareness towards edible insects, but have not had any experience eating them.

**Fig. 3:**
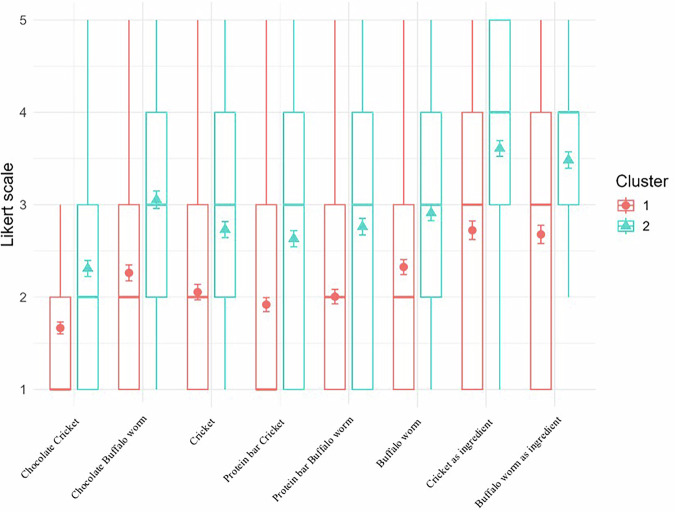
Plot of predicted means (dots) with confidence intervals (error bars) and boxplots by Cluster for different levels of acceptance of insects as food (whole insects—buffalo worm and cricket, and food products—protein bars and chocolate) and appropriateness of using insects as ingredients by Italian children (children = 222; surveys = 444). Acceptance scale of insects as food: 1 = very bad to 5 = very good. Acceptance scale of insects as ingredients: 1 = absolutely no to 5 = absolutely yes.

A significant difference was found between eating behaviour profiles and the levels of acceptance of insects as food (whole insects—buffalo worm and cricket, and food products—protein bars and chocolate) and appropriateness of using insects as food ingredients (Fig. 3). The CLMM revealed that our clusters are significant predictors for WTE insect-based snacks (Protein bar buffalo worm, *E* = 1.1587, *Z*-value = 4.823, and *P*-value = 0.000; Protein Bar Crickets, *E* = 1.1830, *Z*-value = 4.929, and *P*-value = 0.000; Chocolate buffalo worm, *E* = 1.2036, *Z*-value = 5.14, and *P*-value = 0.000; Chocolate Crickets, *E* = 1.2563, *Z*-value = 5.236, and *P*-value = 0.000), whole insects (buffalo worm, *E* = 0.9903, *Z*-value = 4.28, and *P*-value = 0.000; Crickets, *E* = 1.1352, *Z*-value = 4.657, and *P*-value = 0.000), and appropriateness of using insects as food ingredients (buffalo worm, *E* = 1.0318, *Z*-value = 8.533, and *P*-value = 0.000; crickets, *E* = 1.1800, *Z*-value = 5.639, and *P*-value = 0.000). In detail, we observed that *Adventures tasters* were more open to trying these products compared to *Cautious eaters* (Fig. 3).

## Discussion

This study focused on identifying the effect of food neophobia, food disgust, information provision, entomophagy awareness, and eating behaviour profiles on acceptance of insect-based snacks and the moderating role of these traits on educational interventions in Italian school children. The outcomes of the CLMM and latent class analysis showed that food disgust and food neophobia are strong predictors for the acceptance of insect-based snacks, as well as information provision. Moreover, different clusters were found: adventurous tasters and cautious eaters. Interestingly, both low- and moderate-disgust children reported more positive attitudes toward insect-based foods compared with the high-disgust group. The effect was largest for low-disgust children, while moderate-disgust children showed a smaller but still significant positive shift. This pattern may reflect a greater responsiveness of low-disgust children to educational information, or motivational factors such as novelty or social desirability. Understanding these differences is important for tailoring interventions aimed at increasing acceptance of insect-based foods among children with varying levels of food disgust

Overall, neophobic children showed lower food acceptance of all insect-based food snacks. At the same time, children with low or moderate disgust sensitivity showed higher acceptance for all insect-based snacks. These findings align with previous research, which has demonstrated a strong negative correlation between food neophobia and food disgust and the willingness to consume or hedonic ratings of insect-based products^2,15^. Chow et al. argue, based on their research, that disgust may play a more significant role than neophobia among 11- to 13-year-old Danish children due to the perceived animalness of insects^15^. In contrast, Erhard et al. show that food neophobia is negatively associated with the willingness to try insect-based food products among 9- to 13-year-old Danish children, whereas food disgust shows no such relationship^1^. The researchers conclude that disgust sensitivity may not pose a barrier for children, whereas it does for adults. In our research, we found a negative effect of both food neophobia and food disgust on the acceptance of insect-based snacks. The discrepancy between our findings and those of previous research may be attributed to the higher number of children being familiar with insects as food in the study of Erhard et al. In contrast to their study, where over half of the participating children had previously consumed insects, only about ten percent of the children in our study reported prior insect-eating experience. This lack of familiarity with entomophagy among the children in our study may explain why disgust played a more significant role. Additionally, the children in our study were younger than the children in those studies and might therefore have been more disgusted by insects. This is in line with findings by Dupont and Fiebelkorn, who studied the attitude of 9- to 19-year-old German children towards insect burgers^16^. They found that older children show a higher willingness to consume these insect burgers.

Besides food neophobia and food disgust, demographic factors such as age and gender also play a role in the acceptance of insects as food. In our study, males reported significantly higher WTE whole buffalo worm, whole cricket, or cricket chocolate than females. One possible explanation is that males may be less affected by the perceived animalness or visibility of insects than females, as the males assigned higher scores than females to both whole insects and the chocolate bar in which insect components were clearly visible. This pattern is consistent with findings in adult populations, where men show greater acceptance of insect-based food than women^22^. Moreover, age played a role in our study as 10-year-old children assigned higher scores than 8-year-old children to the whole buffalo worm, whole cricket, and buffalo worm chocolate. In line with previous findings, older age can positively contribute to the willingness to consume insect burgers^16^. As children grow older, they seem more interested in foreign or unfamiliar foods as their food repertoire expands. This is due to their growing autonomy, contact with peers outside the family, and their desire to enter the “adult world”^23^. In our study, older children might have viewed eating whole insects as a more grown-up, and therefore more appealing, behaviour than younger children did.

Previous research has mentioned that barriers to consuming insects can be reduced by providing information on the environmental and health benefits of edible insects, and framing insects more positively^12,16^. Our intervention focused on providing children with information about healthy snacking habits, the nutritional value of alternative proteins, and the health and environmental benefits of consuming insects. This was delivered through two educational lectures. We found that educational intervention predicts the WTE of insect-based snacks. Overall, hedonic ratings for insect-based snacks were higher after providing information about the benefits of entomophagy than before, which is in line with findings by Maya et al. on children’s perception of cricket and buffalo worm^2^. This suggests that educating children about the benefits of entomophagy increases their WTE insects, particularly for whole buffalo worm, the buffalo worm protein bar, and the buffalo worm chocolate. In addition, we found that providing educational information attenuates the negative influence of neophobia, indicating that such information can partially offset neophobic responses for certain products, such as the cricket protein bar, but does not reduce the impact of disgust. These patterns highlight the need for further research to clarify when and how educational strategies can effectively improve acceptance of insect-based foods.

Oppositely, educational information in our study did not predict children’s acceptance of whole cricket, the cricket chocolate, or the cricket protein bar. The type of insect used might explain the differences between the buffalo products and the cricket products. Previous studies have shown that children like mealworms more than grasshoppers or crickets, potentially due to their difference in animalness or size^15,17^. Nevertheless, Erhard et al. show that this effect of insect type is eliminated when the insect is processed in a food product, which was not the case in our study^1^. Similarly, van Lier et al. indicate that children were not bothered by eating insect snack balls but were unwilling to try whole crickets^24^. The absence of this elimination effect in our study might be explained by the higher visibility and recognizability of the cricket in all product pictures presented to the children compared to the buffalo worm. Although children showed a stronger preference for mealworms compared to grasshoppers and crickets, this effect was less pronounced among adults^25^. For Dutch adults, WTE was highest for crickets, grasshoppers, and mealworms, likely because these species are most frequently marketed in the Netherlands. However, the differences between these insects were not statistically significant. This could be explained by the fact that this study did not present participants with real insects, which may have reduced the salience of the “animalness” of crickets and grasshoppers.

Providing information about the environmental and health benefits of edible insects may be effective for certain child consumer groups, but not for all. Information given to children about insects should be balanced, as too much information can also have a counterproductive effect^12^. Previous research found that information about the benefits of insects might potentially evoke disgust and therefore does not increase acceptance^1^. Likewise, Nyberg et al. state that information about nutritional and sustainability values is seldom enough; the insect-based products should also be considered appealing as well^12^. Dupont and Fiebelkorn argue that tasting or cooking sessions with insect-based ingredients might be useful for acceptance^16^. Similarly, Hémar-Nicolas argues that entomophagy rejection will not be changed by merely rational arguments, as sensory barriers are key^17^. Tactile interactions with insect-based food can help reduce disgust and arouse curiosity^17^. According to these findings, children should be involved in sensory and participatory activities or cooking programmes. Based on our findings, we argue that these sensory activities might be especially relevant for adventurous tasters. Contrarily, Chow et al. argue that there is no effect of cooking on the liking of insects, possibly because cooking creates higher levels of disgust^15^. This might also apply to the Cautious eaters, who are more easily disgusted. Further research is needed to clarify the effect of sensory activities in WTE insect-based products.

The potential evocation of disgust through information provision could be reduced by increasing familiarity and processing of the insect-based food. Serving insects in a familiar dish rather than as whole insects can increase familiarity with insects in a low-key way and has been shown to increase acceptability^2,12,15,17,24^. Specifically, as shown by Nyberg et al., dishes with insect-based ingredients are perceived as tasty until it is revealed that they contain insects^12^. Children more easily accept insects when the animalness is reduced^17^. Therefore, processed insects, in the form of, for instance, flour, can be incorporated into familiar and well-liked foods. This appears to be an appropriate way to introduce edible insects to the market when familiarity with insect-based food is low^1^.

However, the need to process insect-based foods into familiar meals may vary across different child consumer groups. We found that two groups of children differ in their openness to try insect-based snacks: Cautious eaters and Adventurous tasters. Whereas Cautious eaters have a lower openness to try novel foods and are more likely to be disgusted, Adventurous tasters are more willing to try unfamiliar food, including insect-based foods. These two groups might be targeted in a different way; Adventurous tasters may be motivated to try insect-based foods when consumption is framed as a fun challenge and a novel experience, allowing for greater recognizability of the insect. Conversely, Cautious eaters tend to prefer more familiar foods, so processing insects invisibly within the product may facilitate their acceptance of insect-based foods. Latent class analysis enables the identification of specific eating behaviour profiles among children, laying the groundwork for more targeted educational and marketing strategies. Adventurous tasters might respond better to challenge-based approaches, while cautious eaters may be more open to insect-based foods if the insects are hidden in familiar products. Since childhood is a crucial time for shaping long-term eating habits, these tailored strategies could play an important role in increasing acceptance of insect-based foods early on.

Some limitations of this study include the fact that the sample may not be fully representative of all Italian children. Previous research suggests that southern Italian regions, with deeply rooted food cultures, may be less open to insect-based foods compared with northern regions^26^. Additionally, only a small proportion of participants had previously tried insect-based foods. Future research should replicate these findings in countries or regions where insect consumption is more common. If the educational intervention proves effective, or even more effective, in areas with established entomophagy practices, such as parts of East Asia or South America, this will provide particularly meaningful insights for promoting acceptance of insect-based foods.

The findings of this study are robust within the assumptions regarding acceptance and WTE edible insects-based snacks. The use of realistic snack products, such as chocolate and a protein bar, enhanced the ecological validity of the study and aligned well with current food market trends. The combination of cumulative link (mixed) models and latent class analysis also provided both detailed individual insights and broader group-level findings. Further research into participation motivations could also help identify systematic differences between those who choose to participate and those who do not, thereby shedding light on potential participation bias and improving the generalizability of future study findings. Children completed the survey in classrooms, where peer influence may have biased their responses. Nevertheless, the high participation rate (222 respondents) further supports the reliability of the data.

Future studies could isolate the effect of insect processing by using the same insect type across all conditions. Including a condition with whole insects could also provide a clearer baseline for measuring aversion. To reduce social influence, future research should aim to ensure that children complete surveys individually, ideally in settings that minimize peer interaction. Including actual tasting sessions would provide a more accurate measure of how children’s expectations line up with their real reactions. It would also be useful to test a wider range of insect-based food, including full meals rather than just snacks, and to explore different insect species to uncover more detailed patterns of acceptance. Finally, following children over time in longitudinal studies would help track how their views on edible insects change as they grow and learn more about sustainable food options.

Our analysis suggests that children’s acceptance across various edible insect-based snacks reflects a limited awareness of entomophagy and WTE, with many individuals finding it difficult to accept these types of products. Furthermore, while information provision is often promoted as a tool to drive sustainable-conscious behaviour, our findings indicate that this approach may not be effective for all children. Cultural attachment (food neophobia and food disgust) to traditional food consumption, particularly, poses a substantial barrier to dietary shifts towards sustainability in children.

## Methods

Data were collected by administering a paper-based questionnaire from November 2024 to March 2025. To collect data from children aged 8 to 10, we used a paper-based questionnaire, as this format was considered more suitable for the age group. At this stage of development, many children have limited access to or experience with digital devices, so a paper questionnaire offered a more hands-on, intuitive approach. The questionnaire was written in Italian and initially reviewed by consumer science experts. Once approved, it was pilot tested with a total of three teachers and three children (representing the age group from this study) who were not involved in the study. This pre-test helped identify any unclear wording, missing elements, or potential difficulties participants might face in understanding or answering the questions. Based on their feedback, minor changes were made to improve how the questions and response options were phrased. The final version of the questionnaire (see Supplementary Table) was completed anonymously by the participants. Both children and their parents gave written informed consent to participate after reviewing a project information sheet that explained the study’s purpose. Any data collected from children whose parents did not provide consent were immediately removed during the data cleaning process. The study protocol was reviewed and approved by the Ethics Committee of the University of Turin (Ref: GD/212766/2024). All researchers involved in the project contributed to developing the questionnaire and agreed on its content and the way it would be distributed. The study was carried out in line with the principles of the Declaration of Helsinki.

### Participants

A total of 256 students from local schools in the greater metropolitan area of Turin, Italy, were recruited through Ce.Se.Di. (Centro Servizi Didattici), a regional educational service centre that facilitates access to extracurricular and enrichment activities for schools. Ce.Se.Di. maintains a catalogue of educational initiatives, from which schools can voluntarily select activities to implement in their classrooms. After teachers selected our teaching activity through Ce.Se.Di. catalogue, schools scheduled two sessions *per* class. The second class needed to be scheduled within one week of the first session, coordinated with the research team. Recruitment for the study was open to teachers of 3rd, 4th, and 5th-grade classes, all of whom were teaching in physical classrooms at the time of the study. This age group was selected because children from the age of 7 typically develop sufficient language and reading skills, allowing them to complete the test independently^1^. Participants came from seven different schools and a total of 12 different classes. Participation was voluntary, and inclusion criteria were being 8–10 years old, being fluent in Italian, and living in the metropolitan area of Turin, Italy. Exclusion criteria include incomplete data and lack of parental consent to use data for research (*n* = 36).

### Study design and questionnaire

This study used a quantitative method based on self-reported questionnaires. It followed a within-subjects design with repeated measures, meaning each child’s WTE insect-based snacks and their reactions to them were measured and compared over time. Two sessions took place, and each session included an interactive lecture and a 15-question survey. The lectures were delivered in Italian by one of two Italian-speaking researchers. The questionnaires, also in Italian, were filled out by the children on their own, though they could ask for help from the researchers or their teachers if they needed it.

The study took place over two sessions, each lasting about two hours. Every session included a 75–90-min interactive lecture supported by PowerPoint slides, broken into 15–18-min segments, followed by hands-on activities to keep the children engaged. In the first session, children began by completing the questionnaire (Supplementary Table), then participated in a session focused on protein and healthy snack options, accompanied by related interactive tasks. The focus on protein and healthy snacks in the first lectures was made to draw a baseline understanding of the main nutritional component of edible insects and the important role of proteins for body function. The second session started with a lecture on insects as a food source and their role in sustainability, again followed by interactive activities. This session ended with the children filling out the same questionnaire again.

All the content presented in the lectures was neutral and based on peer-reviewed, scientifically validated research. If any information came from sources like chefs rather than scientific studies, it was thoroughly fact-checked. Throughout the lectures, images of insects, people eating insects, and insect-based dishes were shown to give students a visual sense of the topic. To keep the children engaged and reinforce what they were learning, the sessions also included interactive games as attention checks. The questionnaire and lecture content were originally created in Italian by native speakers. All materials underwent pre-testing to ensure clarity and consistency prior to their use in the study. Although the study took place in a classroom setting, each child completed the tasks individually.

The questionnaire was administered twice, with a one-week interval, for the same participants to assess short-term changes. A total of 256 questionnaires were completed (88% response rate). Twelve percent of the sample (*n* = 34) were excluded due to incomplete data. The final analysis included data from 222 children. The average time to complete the survey was approximately 30–40 min. The questionnaire included the following measures:

- *Willingness to eat* an insect-based snack in the future was measured using a 5-point Likert scale (ranging from 1 = absolutely no to 5 = absolutely yes) to investigate children’s readiness to consume insect-based products.

- *Food neophobia test tool:* this validated scale was composed of ten items using a 5-point Likert scale (ranging from 1 = strongly disagree to 5 = strongly agree)^27^. Cronbach’s Alpha was equal to 0.82 (raw alpha = 0.81, average inter-item correlation = 0.31).

- *Food disgust scale*: this validated scale was composed of eight items using a 5-point Likert scale (ranging from 1 = very disgusting to 5 = very pleasant)^28^. Cronbach’s Alpha was equal to 0.76 (raw alpha 0.75, average inter-item correlation = 0.31).

- *Experience awareness*: this self-constructed scale composed of one item to assess awareness of the existence of and experience with edible insects using a 3-point Likert scale (ranging from 1 = I did not know, 2 = I know, but I haven’t tasted, and 3 = I know, and I have tasted)^1^.

- *Attitudes toward insect-based snacks*: participants were shown two images (one of a buffalo worm and one of a cricket), both in their dried and frozen forms. They were asked to share how appealing they found each insect, and how suitable they thought each was as an ingredient for human food, using a 5-point Likert scale (ranging from 1 = very bad to 5 = very good), with appropriate anchors.

After that, they were shown images of four different insect-based snacks available on the market: two types of protein bars, two kinds of chocolate. All of these products were made using either buffalo worm or cricket flour, and only one product contained visible insect parts. For each item, it was clearly stated which type of insect flour had been used. In total, a five-item scale was developed, each corresponding to different insect-based snacks (two cricket-based snacks, two buffalo worm-based snacks and two edible insects), to evaluate attitudes using a 5-point Likert scale (ranging from 1 = very bad to 5 = very good), with appropriate anchors ^1^.

- *Socio-demographic characteristics* (gender, age, house composition, parental employment status)

### Statistical analysis

An a priori power analysis using the *pwr* package (multiple regression approximation, six predictors, Cohen’s *f* = 0.40, *α* = 0.05, 1 − *β* = 0.80) indicated a minimum sample of *N* ≈ 52. The final sample of 222 children far exceeded this, providing adequate power. Because this approximation does not fully account for the repeated-measures ordinal design, we also conducted a simulation-based power analysis using 500 datasets with the same sample size, repeated measures’ structure, and plausible parameter values for fixed effects and random intercepts (SD = 0.8). For each dataset, a CLMM was fitted, and the proportion of simulations in which the educational effect was statistically significant (*α* = 0.05) was calculated. The estimated empirical power was ≥0.80, confirming that the study was sufficiently powered to detect the expected effects.

Cumulative Link Mixed Models (CLMM) were applied to examine how variables such as food neophobia tool score (FNTT), food disgust scale (FDS), educational information, entomophagy awareness, gender, and age relate to children’s food-related behaviours, which were measured using an ordinal scale. Treating FDS and FNTT as a continuous factor created an excessive number of levels and interaction parameters, resulting in a non-identifiable CLMM (NaN standard errors, singular Hessian). Because the model could not estimate stable coefficients, the continuous specification was not statistically valid. CLMM was selected due to its suitability for ordered categorical data and its ability to account for both fixed effects (educational information, FNTT, FDS, entomophagy awareness and eating behaviour profiles) and random effects (individual participant), thus addressing the within-subjects design of the study. The *ordinal* package was used to fit the models, and model fit was evaluated using likelihood-ratio tests and the Akaike Information Criterion (AIC). The analysis aimed to assess whether the hypothesized relationships among the variables were supported by the data. The model identified which variables significantly predicted the outcome and the direction of their effects, indicating whether they increased or decreased the likelihood of selecting higher categories on the response scale. All statistical analyses were performed using R (version 4.4.2, The R Foundation for Statistical Computing Platform). Descriptive statistics, including means, standard deviations, and frequency distributions, were calculated to support interpretation. Assumptions underlying each model were assessed, and residual diagnostics were performed to ensure the validity and reliability of the results.

The frequency distribution of FNTT was calculated and the subjects were divided into the three following groups: “neophilic” (subjects in the lowest quartile, FNTT ≤ 25, *n* = 25), “moderate neophobia” (subjects in the second and third quartile, FNTT ≥ 26 and ≤35, *n* = 125) and “neophobic” (subjects in the highest quartile, FNTT ≥ 36, *n* = 72). The frequency distribution of FDS was calculated, and the subjects were divided into three following groups: “low disgust” (subjects in the lowest quartile, FDS ≤ 18, *n* = 12), “moderate disgust” (subjects in the second and third quartile, FDS ≥ 19 and ≤28, *n* = 64) and “high disgust” (subjects in the highest quartile, FDS ≥ 29, *n* = 146). Cronbach’s alpha was used to assess the internal consistency of the construct scales, where a value greater than 0.70 is usually recommended using the *alpha* function from the *psych* package in R. Random effects for class and school differences did not significantly add to the model and were therefore deleted from all mixed models.

To identify unobserved subgroups based on shared response patterns (eating behaviour profiles), LCA was conducted using the poLCA package. Model selection was guided by the Bayesian information criterion, entropy values, and the interpretability of the resulting classes. The number of latent classes was determined by balancing statistical fit and conceptual clarity. A significance level of *α* = 0.05 was applied to all hypothesis testing.

## Supplementary information

Supplementary Information

## Data Availability

The data sets used and/or analysed during the current study are available from the corresponding author upon request.
