# Supplementary material for: Predicting schoolchildren’s willingness to eat insect-based snacks: effects of information provision psychological and cognitive traits
Source: NPJ Sci Food. 2026 Apr 8;10:179. doi: 10.1038/s41538-026-00826-3 (PMC13237090; doi:10.1038/s41538-026-00826-3)
Supplement: Supplementary file 1 — Supplementary Information [file 41538_2026_826_MOESM1_ESM.docx]

**Supplementary Tables**

**Supplementary Table 1.** Survey in English and Italian language

**Number/Numero_________________________**

**Questionare**

1. Rispondi alla seguente domanda barrando la casella sotto l’affermazione che preferisci. (Answer the following question by ticking the box below the statement you prefer)

|  | 1 | 2 | 3 | 4 | 5 |
| --- | --- | --- | --- | --- | --- |
|  | Assolutamente no (Absolutely no) | No | Non credo  (Uncertain) | Sì  (Yes) | Assolutamente sì (Absolutely yes) |
| Mangeresti uno spuntino confezionato a base di insetti in futuro? (Would you eat a snack made from insects in the future?) |  |  |  |  |  |

* Erhard *et al.,* 2023

1. Rispondi alla seguente domanda barrando la casella sotto l’affermazione che preferisci. (Answer the following question by ticking the box below the statement you prefer)

|  | 1 | 2 | 3 | 4 | 5 |
| --- | --- | --- | --- | --- | --- |
|  | Per niente d’accordo (Strongly disagree) | Disaccordo (Disagree) | Indeciso (Uncertain) | D’accordo (Agree) | Molto d’accordo (Strongly agree) |
| 1. Mi piace provare cibi che non ho mai assaggiato prima. (I like to try foods I have never tasted before.) |  |  |  |  |  |
| 2. Mi piace provare cibi nuovi e diversi. (I like to experience new and different foods.) |  |  |  |  |  |
| 3. Penso che sia divertente provare cibi che non conosco. (I think it is fun to try food items I don't know.) |  |  |  |  |  |
| 4. Proverei del cibo anche se non so cosa sia. (I will try food even though I don't know what it is.) |  |  |  |  |  |
| 5. Mi piacenno diversi tipi di cibi. (I enjoy a wide variety of different foods.) |  |  |  |  |  |
| 6.Non ho paura di mangiare cose che non ho mai assaggiato o provato prima. (I am not afraid of eating things I have not tasted or experienced before.) |  |  |  |  |  |
| 7. Non mi dispiace mangiare cibi a cui non sono abituato. (I don't mind eating foods I am not used to.) |  |  |  |  |  |
| 8. Penso che il cibo sconosciuto sembri poco appetitoso. (I think unfamiliar food looks unappetizing.) |  |  |  |  |  |
| 9. Ho paura a provare cibi che non ho mai assaggiato prima. (I am afraid of trying food I have not tasted before.) |  |  |  |  |  |
| 10. Sono disposto ad assaggiare cibi preparati con insetti. (I am willing to taste foods made with insects.) |  |  |  |  |  |

* Erhard *et al.,* 2023

1. Rispondi alla seguente domanda barrando la casella sotto l’affermazione che preferisci. (Answer the following question by ticking the box below the statement you prefer)

|  | 1 | 2 | 3 | 4 | 5 |
| --- | --- | --- | --- | --- | --- |
|  | Molto gradevole (Very pleasant) | Abbastanza gradevole (Pleasant) | Indeciso (Uncertain) | Abbastanza disgustoso (Disgusting) | Molto disgustoso (Very disgusting) |
| 1. Mangiare con le posate sporche in un ristorante. (To eat with dirty silverware in a restaurant.) |  |  |  |  |  |
| 2. Mangiare cibo ricevuto da un vicino che conosco poco. (Food donated from a neighbor whom I barely know.) |  |  |  |  |  |
| 3. Mangiare formaggio duro (per esempio Parmigiano) da cui è stata tolta la muffa. (To eat hard cheese from which mold was cut off.) |  |  |  |  |  |
| 4. Mangiare fette di mela diventate marroni perché esposte all'aria. (To eat apple slices that turned brown when exposed to air.) |  |  |  |  |  |
| 5. La consistenza di alcuni tipi di pesce in bocca. (The texture of some kinds of fish in the mouth.) |  |  |  |  |  |
| 6. Mangiare la polpa di avocado di colore marrone. (To eat brown-coloured avocado pulp.) |  |  |  |  |  |
| 7. Mangiare insalata in cui c’è una lumaca. (There is a little snail in the salad that I wanted to eat.) |  |  |  |  |  |
| 8. Mettermi in bocca i tendini di un animale (i tendini sono le parti che collegano i muscoli alle ossa). (To put animal tendons into my mouth). |  |  |  |  |  |

*Hartmann *et al.,* 2018

1. Quanto ne sai degli insetti come cibo? (How much do you know about insects as food?)

Scegli UNA delle opzioni seguenti (Please choose ONE of the options below)

☐ Non sapevo fossero cibo (Didn’t know it was food)

☐ So che è cibo, ma non l'ho mai assaggiato (Know it is food, but have never tasted)

☐ So che è cibo e l'ho assaggiato (Know it is food and have tasted)

Due degli insetti più disponibili sul mercato in Italia sono il verme Bufalo worm e i grilli. Questa immagine qui sotto è di un verme chiamato Bufalo worm essiccato (Two of the most available insects on the market in Italy are buffalo worms and crickets. This picture below is of a buffalo worm)


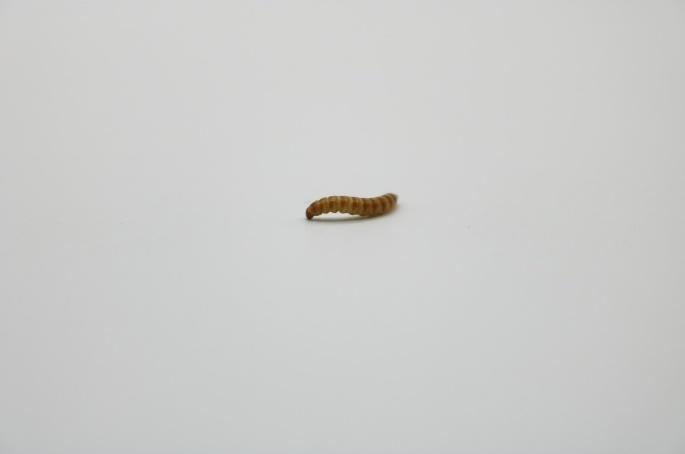


|  | Molto male  (Very bad) | Male  (Bad) | Neutro  (Neutral) | Bene  (Good) | Molto bene  (Very good) |
| --- | --- | --- | --- | --- | --- |
| Come ti senti guardando il Bufalo worm? How do you feel looking at the buffalo worm?) |  |  |  |  |  |

1. Rispondi alle seguenti domande barrando la casella sotto l’affermazione che preferisci (Answer the following question by ticking the box below the statement you prefer)

|  | 1 | 2 | 3 | 4 | 5 |
| --- | --- | --- | --- | --- | --- |
|  | Assolutamente no (Absolutely no) | No | Non credo (Uncertain) | Sì (Yes) | Assolutamente sì (Absolutely yes) |
| Pensi che i bufalo worm possano essere usati come ingrediente alimentare? (Do you think that buffalo worms can be used as a food ingredient?) |  |  |  |  |  |


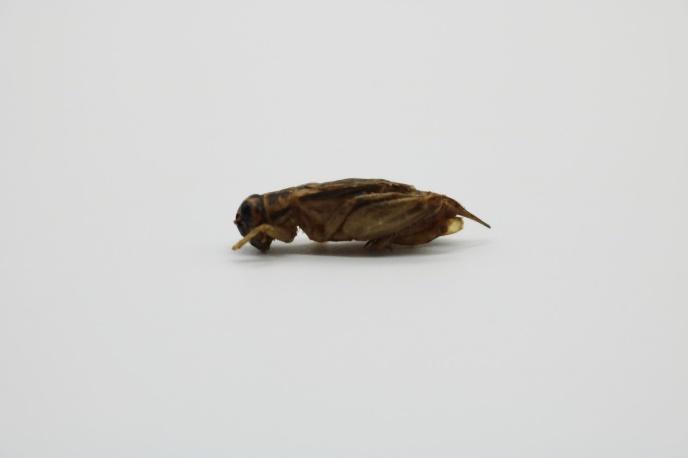
*Questa immagine qui sotto è di un grillo essiccato (This picture below is of a cricket)*

1. Scegli UNA delle opzioni seguenti (Please choose ONE of the options below)

|  | Molto male  (Very bad) | Male  (Bad) | Neutro  (Neutral) | Bene  (Good) | Molto bene  (Very good) |
| --- | --- | --- | --- | --- | --- |
| Come ti senti guardando il grillo? How do you feel looking at the cricket?) |  |  |  |  |  |

1. Rispondi alle seguenti domande barrando la casella sotto l’affermazione che preferisci (Answer the following question by ticking the box below the statement you prefer)

|  | 1 | 2 | 3 | 4 | 5 |
| --- | --- | --- | --- | --- | --- |
|  | Assolutamente no (Absolutely no) | No | Non credo (Uncertain) | Sì (Yes) | Assolutamente sì (Absolutely yes) |
| Pensi che i grilli possano essere usati come ingrediente alimentare? (Do you think that crickets can be used as a food ingredient?) |  |  |  |  |  |

*Ora vedrai 4 diverse merende ricche di proteine, tutte realizzate con farina di insetti. Alcuni alimenti sono fatti con farina di Bufalo worm e altri con farina di grillo (Now, you will see 4 different protein-rich snacks that are all made with insect flour. Some foods are made with buffalo worm flour and others are made with cricket flour)*

*Questa barretta proteica è realizzata con farina di Bufalo worm (This protein bar is made with buffalo worm flour)*


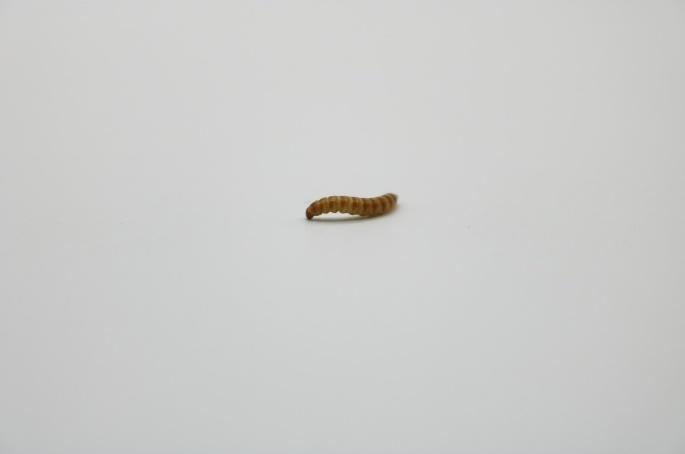

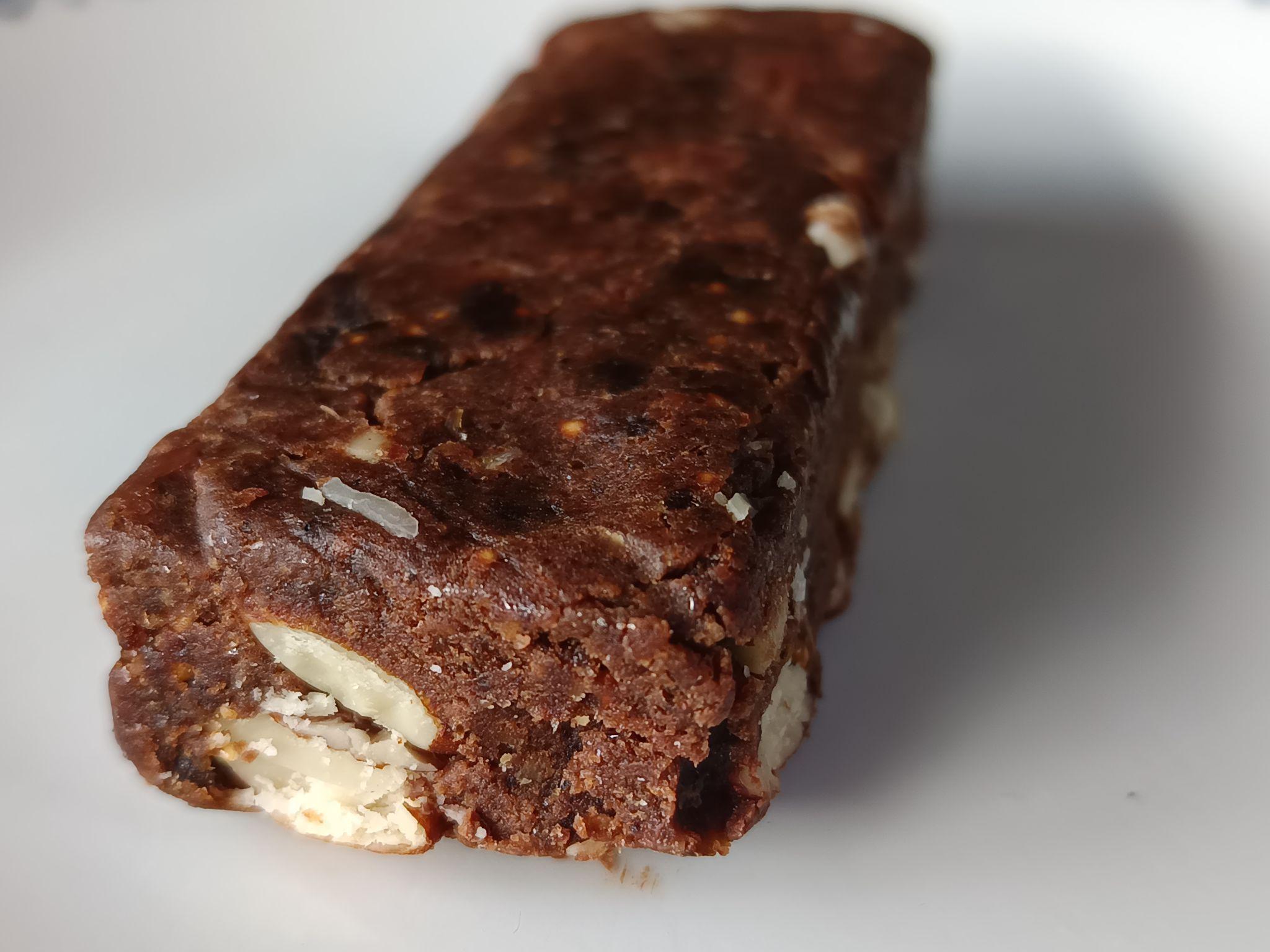


1. Scegli UNA delle opzioni seguenti (Please choose ONE of the options below)

|  | Molto male  (Very bad) | Male  (Bad) | Neutro  (Neutral) | Bene  (Good) | Molto bene  (Very good) |
| --- | --- | --- | --- | --- | --- |
| Come ti senti all’idea di assaggiare questa barretta proteica fatto con farina di Bufalo worm? (How do you feel about tasting this protein bar?) |  |  |  |  |  |

*Questo cioccolato è fatto con farina di Bufalo worm (This chocolate is made with buffalo worm flour)*


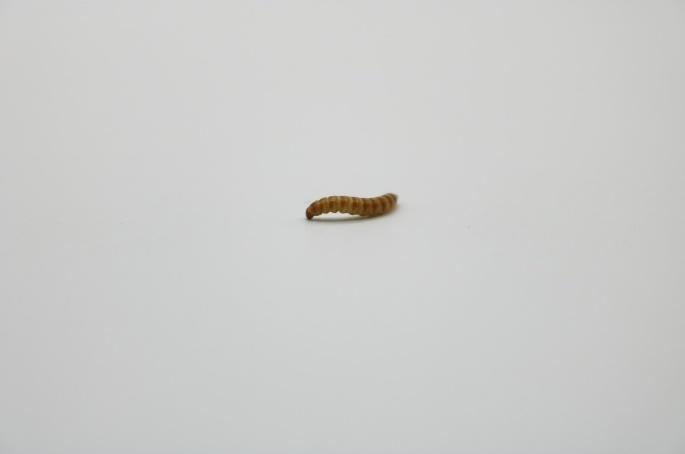

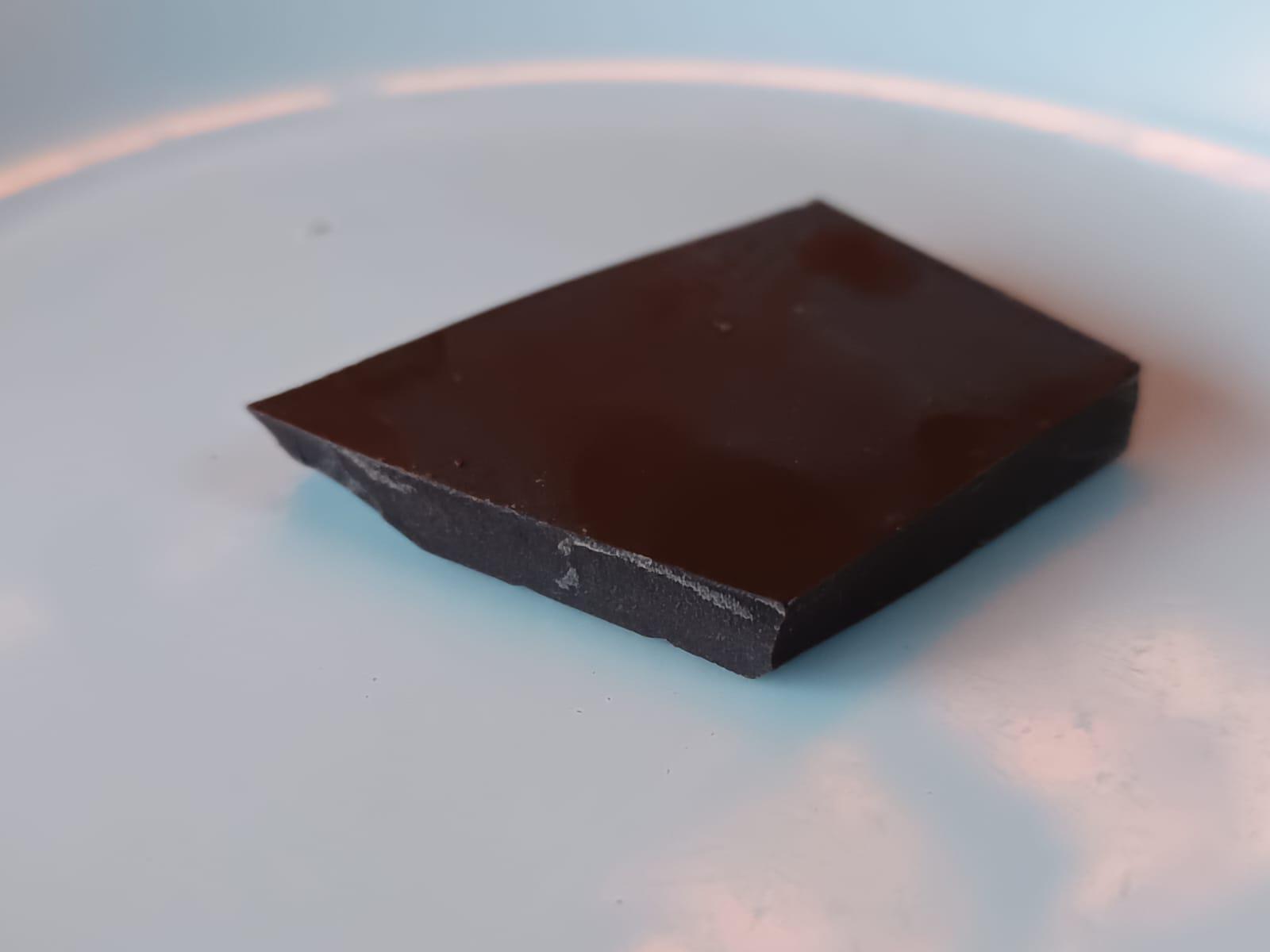


1. Scegli UNA delle opzioni seguenti (Please choose ONE of the options below)

|  | Molto male  (Very bad) | Male  (Bad) | Neutro  (Neutral) | Bene  (Good) | Molto bene  (Very good) |
| --- | --- | --- | --- | --- | --- |
| Come ti senti all’idea di assaggiare questo cioccolato fatto con farina di Bufalo worm? (How do you feel about tasting the chocolate buffalo worm? |  |  |  |  |  |

*Questa barretta proteica è fatta con farina di grillo (This protein bar is made with cricket flour)*


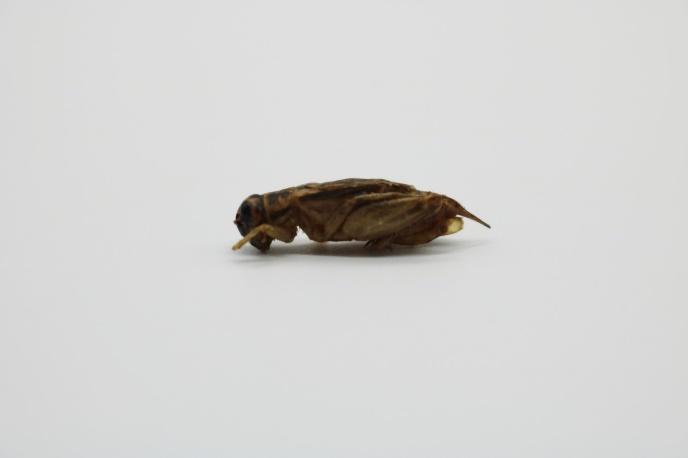

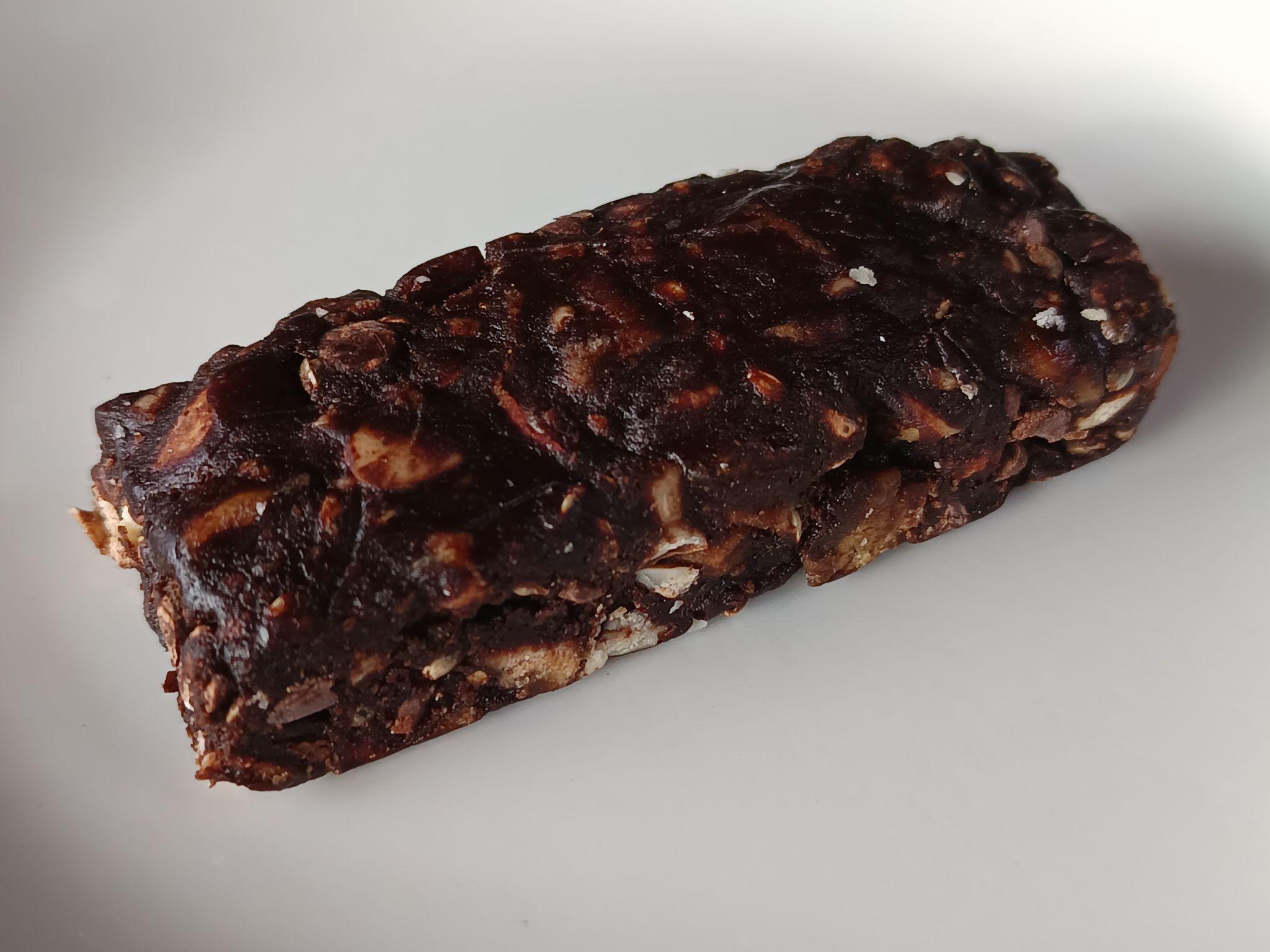


1. Scegli UNA delle opzioni seguenti (Please choose ONE of the options below)

|  | Molto male  (Very bad) | Male  (Bad) | Neutro  (Neutral) | Bene  (Good) | Molto bene  (Very good) |
| --- | --- | --- | --- | --- | --- |
| Come ti senti all’idea di assaggiare questa barretta proteica fatto con farina di grillo? (How do you feel about tasting the protein bar cricket?) |  |  |  |  |  |

*Questo cioccolato è fatto con farina di grillo (This chocolate is made with cricket flour)*


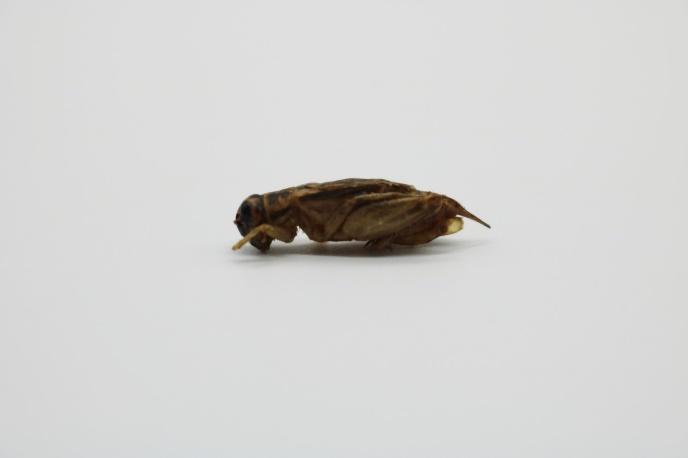

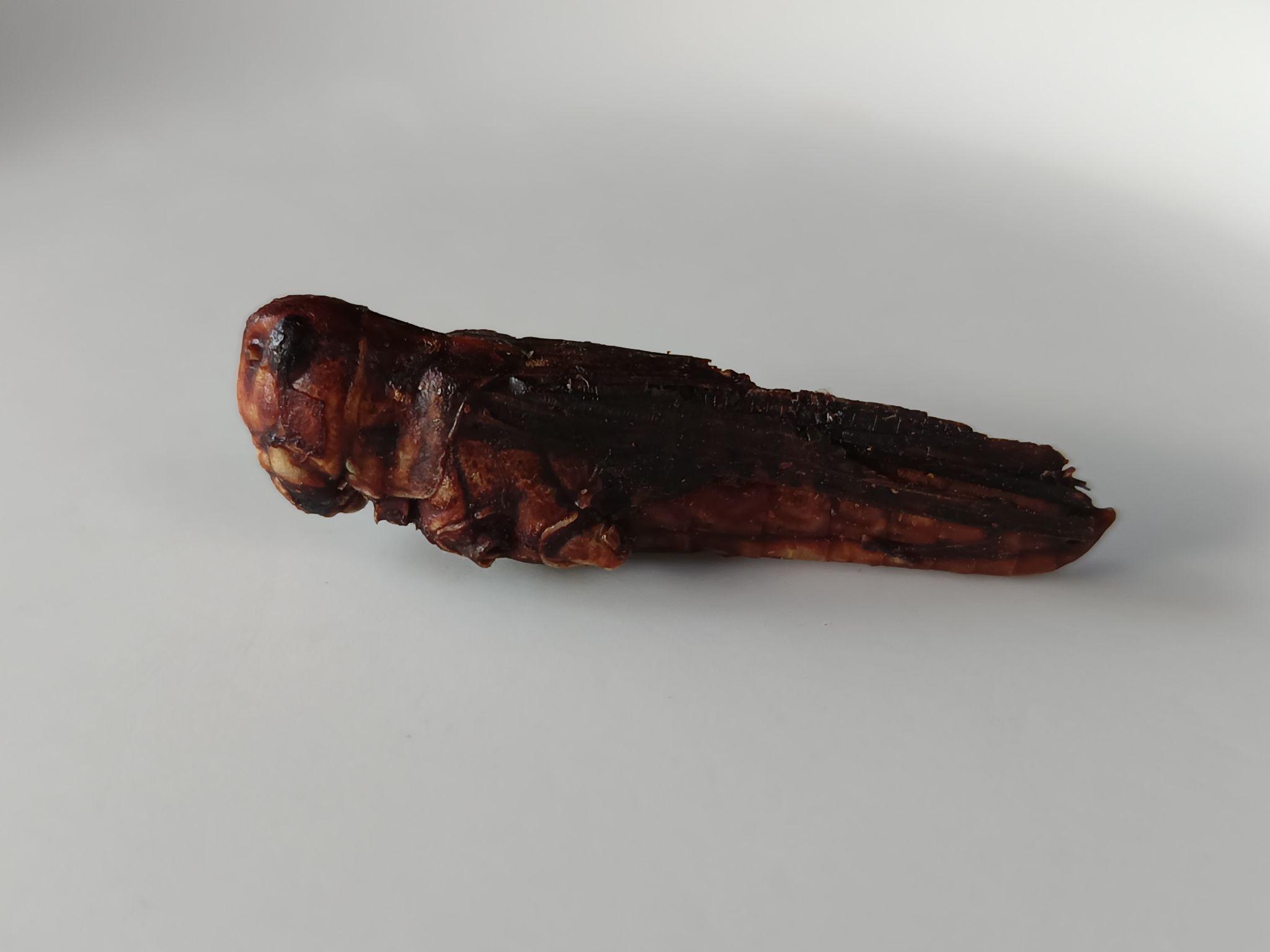


1. Scegli UNA delle opzioni seguenti (Please choose ONE of the options below)

|  | Molto male  (Very bad) | Male  (Bad) | Neutro  (Neutral) | Bene  (Good) | Molto bene  (Very good) |
| --- | --- | --- | --- | --- | --- |
| Come ti senti all’idea di assaggiare questo cioccolato fatto con farina di grillo? How do you feel about tasting the chocolate cricket?) |  |  |  |  |  |

1. Quanti anni hai? (How old are you?)

Scegli UNA delle opzioni seguenti (Please choose ONE of the options below)
☐ 9
☐ 10
☐ 11

1. Sei una femmina o un maschio? (Are you a boy or a girl?)

Scegli UNA delle opzioni seguenti (Please choose ONE of the options below)
☐ Femmina (Girl)
☐ Maschio (Boy)

1. Con chi vivi? (Who do you live with?)

Scegli UNA delle opzioni seguenti (Please choose ONE of the options below)
☐ Entrambi genitori *(Both parents)*
☐ Solo mamma (mother only)
☐ Solo papà (father only)
☐ Tutori (guardians*)*

1. I tuoi genitori lavorano? (Do your parents work?)

Scegli UNA delle opzioni seguenti (Please choose ONE of the options below)
☐ Sì, entrambi lavorano (Both parents)
☐ Solo mamma (mother only)
☐ Solo papà (father only)
☐ No, sono entrambi disoccupati (both unemployed*)*
☐ Preferisco non dire / Non so *(Prefer not to say/Don’t know)*
